# Understanding Calmodulin Variants Affecting Calcium-Dependent Inactivation of L-Type Calcium Channels through Whole-Cell Simulation of the Cardiac Ventricular Myocyte

**DOI:** 10.3390/biom13010072

**Published:** 2022-12-29

**Authors:** Matthew D. McCoy, Aman Ullah, W. Jonathan Lederer, M. Saleet Jafri

**Affiliations:** 1 School of Systems Biology, George Mason University, Fairfax, VA 22030, USA; 2 Innovation Center for Biomedical Informatics, Department of Oncology, Georgetown University Medical Center, Georgetown University, Washington, DC 20057, USA; 3 Center for Biomedical Engineering and Technology, University of Maryland School of Medicine, Baltimore, MD 20201, USA

**Keywords:** CPVT4, LQT14, calmodulin, arrhythmia, heart, LQT, CPVT

## Abstract

Mutations in the calcium-sensing protein calmodulin (*CaM*) have been linked to two cardiac arrhythmia diseases, Long QT Syndrome 14 (LQT14) and Catecholaminergic Polymorphic Ventricular Tachycardia Type 4 (CPVT4), with varying degrees of severity. Functional characterization of the *CaM* mutants most strongly associated with LQT14 show a clear disruption of the calcium-dependent inactivation (CDI) of the L-Type calcium channel (LCC). CPVT4 mutants on the other hand are associated with changes in their affinity to the ryanodine receptor. In clinical studies, some variants have been associated with both CPVT4 and LQT15. This study uses simulations in a model for excitation–contraction coupling in the rat ventricular myocytes to understand how LQT14 variant might give the functional phenotype similar to CPVT4. Changing the *CaM*-dependent transition rate by a factor of 0.75 corresponding to the D96V variant and by a factor of 0.90 corresponding to the F142L or N98S variants, in a physiologically based stochastic model of the LCC prolonger, the action potential duration changed by a small amount in a cardiac myocyte but did not disrupt CICR at 1, 2, and 4 Hz. Under beta-adrenergic simulation abnormal excitation–contraction coupling was observed above 2 Hz pacing for the mutant *CaM*. The same conditions applied under beta-adrenergic stimulation led to the rapid onset of arrhythmia in the mutant *CaM* simulations. Simulations with the LQT14 mutations under the conditions of rapid pacing with beta-adrenergic stimulation drives the cardiac myocyte toward an arrhythmic state known as Ca^2+^ overload. These simulations provide a mechanistic link to a disease state for LQT14-associated mutations in *CaM* to yield a CPVT4 phenotype. The results show that small changes to the *CaM*-regulated inactivation of LCC promote arrhythmia and underscore the significance of CDI in proper heart function.

## 1. Introduction

Calmodulin (*CaM*) mutants have been associated with cardiac arrhythmia in clinical and experimental studies [1,2,3]. *CaM* mutants that disrupt the Ca^2+^-dependent inactivation (CDI) of the L-type Ca^2+^ channel have been associated in experimental studies with LQT14 [1,3]. *CaM* variants that have reduced affinity for the ryanodine receptor and increase RyR open probability have been associated experimentally with CPVT4 [1,3]. However, in clinical studies, associating variants with disease is more difficult. The D96V and N98S *CaM* variants have been associated with both LQT14 and CPVT4 [4,5,6,7,8]. This study uses a computational model for EC coupling in the rat ventricular myocyte to study how reductions in CDI similar to those seen in the N98S and D96V mutations can lead to Ca^2+^ overload and therefore be confused with the CPVT4 phenotype. 

In the cell, networks of individual protein interactions combine to produce emergent cell behavior. Discovering how proteins function in the cell is a true scientific endeavor, where experiments are built to test and expand the understanding of these molecular systems. The iterative undertaking requires building up mechanistic theory through carefully designed and executed experimentation but is limited by the cost of execution and biological complexity. Computational modeling provides a platform to integrate the previous knowledge of these complex systems, where competing theories can be explored and expanded [9,10]. Models which faithfully reproduce emergent behavior are useful in testing the sensitivity of the phenotype to the individual interactions within a system and can be used to direct the course of future experiments. The growing accessibility and power of computational resources enable more complex modeling to be realized, and the pairing with experimental studies will continue to become commonplace.

**Figure 1:**
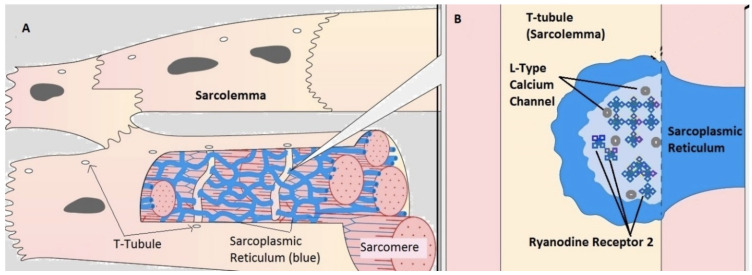
Cardiac myocyte structure. In the cardiac myocyte (**A**), CICR occurs at a specialized sub-domain called a dyad (**B**). The array of L-type calcium channels (LCC) on the T-tubule membrane and ryanodine receptor 2 (RYR2) channels on the sarcoplasmic reticulum (SR) make up the CRU.

A powerful application of cellular models is the ability to test a variety of underlying conditions which lead to similar emergent behavior and has been very successful exploring the process of excitation–contraction (EC) coupling in the cardiac myocyte [11,12]. These excitable cells respond to an action potential by initiating a contractile stimulating release of calcium from sarcoplasmic reticulum (SR) [13]. The cellular structure of the cardiac myocyte (Figure 1A) contains specialized calcium release structures known as Calcium Release Units (CRU), where the SR is closely associated with the T-tubule (Figure 1B). When the cell membrane is depolarized, L-type Calcium Channels (LCC) open and allow calcium to enter the small junctional volume. Ryanodine Receptor 2 (RYR2) channels on the surface of the SR membrane open in response to allow the flow of additional calcium from the internal store in a process known as CICR. 

Central to EC coupling in the cardiac ventricular myocyte Ca^2+^ are the elementary events that give rise to the Ca^2+^ transient consists of many sparks [14]. Under conditions of high intracellular Ca^2+^ referred to as Ca^2+^ overload, spontaneous Ca^2+^ release observed. Under high resolution, spontaneous release can be observed Ca^2+^ waves which consist of the summation of sparks to create a propagating wave [15,16]. When spontaneous Ca^2+^ release, which consists of the summation of Ca^2+^ sparks, occur at several subcellular locations this can result in an arrhythmia membrane depolarization [15,17]. Many studies have shown the link between spontaneous Ca^2+^ release, propagation of Ca^2+^ release, and the resulting membrane depolarization on both the spatial and non-spatial scales [15,18]. Non-spatial models are useful for understanding cellular dynamics in ventricular myocytes as has been shown in many published papers [19]. Spatial models while useful in understand Ca^2+^ dynamics and spontaneous Ca^2+^ waves are very computationally expensive and have seem limited used to understand EC coupling in simulations involving pacing for many seconds. 

Following CICR, the cell is returned to a diastolic state by various ion pumps, restoring cellular ionic concentrations to their initial values. Many proteins play a role in maintaining the cycle, each providing an opportunity to produce an emergent arrhythmia disease phenotype through its altered function. The disease is complex with a large number of different disease variants attributed to mutations in different proteins and is illustrated by the naming conventions for two of these diseases, Long QT Syndrome 14 (LQT14) and catecholaminergic polymorphic ventricular tachycardia Type 4 (CPVT4). Characterized by an elongation of the QT interval on the electrocardiogram, the thirteen LQT14 variants arise out of functional abnormalities in different proteins responsible for restoring the transmembrane voltage after depolarization [20,21,22]. The CPVT4 phenotype is more difficult to diagnose, as fatal arrhythmia is present only under cardiac stress [23]. By integrating the individual contributions of each component in the system, computational models can be used to identify novel conditions which may give rise to disease. This is especially true for the cardiac myocyte, where similar cell behavior can arise despite different origins in electrical handling or calcium release.

The recent association of mutations in the calcium binding protein *CaM* with LQT14 and CPVT4 blur the line between the disease classifications [24,25]. The sequence variations, shown in Table 1, have been characterized through several independent studies [8,26,27,28,29,30,31,32,33]. Most of the mutations occur near one of the *CaM* EF-hand Ca^2+^ binding domains and disrupt Ca^2+^ binding to varying degrees. Several of the mutants have been associated with varying severity of LQT14 or CPVT4, while others have been reported to show characteristics of both (e.g., N98S). *CaM* acts to convey calcium signals in the cell and interacts with multiple proteins involved in regulating EC coupling. For some mutants, the impact on LCC Ca^2+^-dependent inactivation and RYR2 open probability has been reported, but it remains unclear how certain mutations can present as both LQT14 and CPVT4 [31,32]. In addition to the direct interaction with ion channels, differences in gene expression of other system components and subcellular geometry could influence the emergence of an arrhythmic prone state.

Gene variants are classified as being associated with a disease phenotype through clinical studies and GWAS (Genome Wide Association Studies). Genetic analysis can be a powerful tool when used as a marker for hard to diagnose conditions, but harmful mutations can be mischaracterized by failing to consider the systematic impact of small changes to individual molecular interactions. In the case of LQT14, *CaM* mutations which do not inflict a detectable impact on the action potential may be dismissed as benign. Classified as less severe and associated with harder to diagnose conditions, dismissing their role in Ca^2+^-dependent inactivation (CDI) eliminates the consideration of therapies targeted at LCC regulation. Using a computational model of EC coupling, the impact of mutant *CaM* on individual interactions can be examined in detail to make inferences about the mechanisms underlying emergent cell behavior. By demonstrating how even a fractional disruption in a key component of CICR can increase susceptibility to fatal arrhythmia, computational simulations provide a deeper understanding the link between sequence variation and disease. These insights can be used to support mechanistic hypotheses and motivate continued research into complex genetic disease.

## 2. Materials and Methods

### 2.1. Compartmental Cellular Model

**Figure 2:**
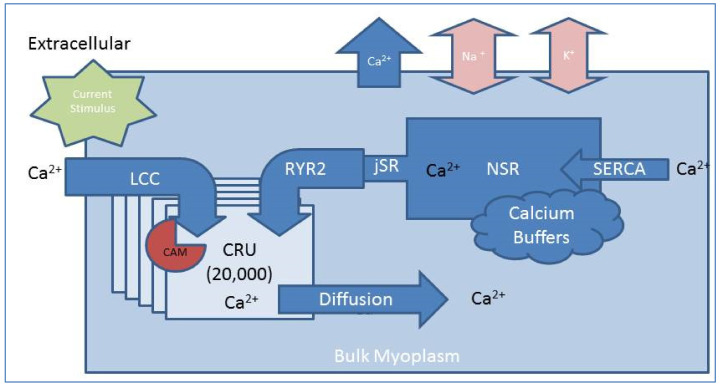
Compartmental model of the cardiac myocyte. A schematic representation of the cardiac myocyte compartmental model, which is parameterized using single channel experimental data, is used to simulate the cellular ionic flux. Calcium enters the cell through 20,000 independent CRUs, first by the L-Type Calcium Channel (LCC) in response to a current stimulus and then through Ryanodine Receptor 2 (RYR2) channels on the surface of the junctional Sarcoplasmic Reticulum (jSR). Calcium influx is terminated by a process dominated by *CaM*-dependent inactivation the LCC. Calcium diffuses into the bulk myoplasm, where it is reloaded into the non-junctional Sarcoplasmic Reticulum (NSR) by sarcoplasmic/endoplasmic reticulum Ca^2+^-ATPase (SERCA) to diffuse back to the jSR. Additionally, calcium buffers in the bulk myoplasm and NSR account for calcium interactions outside the scope of CICR and cellular ionic homeostasis is maintained by calcium, sodium, and potassium exchangers in the cell membrane.

A physiologically realistic, compartmental model built to understand the intricacies of calcium dynamics in the CRU of was expanded to explore the impact of deficiencies in *CaM* regulated inactivation of LCC in a computational model of the rat cardiac ventricular myocyte [34]. The model, shown schematically in Figure 2, models the CICR from the SR through the stochastic simulation of 20,000 independent CRUs. In each of the 20,000 CRUs, there are 49 RyR2s and 7 LCC. Experimental studies have shown that there are 30–300 RyR2s in the cardiac dyad. Our previous studies showed that in this range the spark properties do not change much in the range of 50–300 RyR2 channels [35]. Forty-nine channels were chosen because it was easier to derive the mean field coupling between RyRs in this square array [36].

Our model presented in [1] includes all the membrane currents and improves upon our previous work that focused solely on the Ca^2+^ dynamics [2]. The opening probability of each LCC channel on the T-tubule membrane is dependent on the transmembrane voltage and opens in response to the injection of current via a simulated current clamp protocol. The RYR2 channels open in response to the flow of calcium into the small volume of the dyadic subspace, releasing calcium from the SR to diffuse into the bulk myoplasm. The increase in Ca^2+^ in the dyad also triggers the *CaM*-dependent inactivation of the LCC to halt the flow of extracellular calcium into the cell. The bulk of the released calcium is reloaded back into the SR via sarcoplasmic/endoplasmic reticulum Ca^2+^-ATPase (SERCA) pumps, and ionic homeostasis is achieved as Ca^2+^, Na^+^, and K^+^ pumps in the membrane restore the transmembrane potential. Additional interactions of calcium in both the cell and SR are accounted for by buffers in the myoplasm and SR.

#### 2.1.1. Calcium Release Site (CRU)

A calcium release site is formed by the dyadic subspace and contains a cluster of 49 RyR2 channels and 7 LCCs channels. At each calcium release site, dynamic calcium buffering is implemented for three different endogenous buffers: calmodulin (*CaM*), sarcolemmal (SL) buffer and sarcoplasmic reticulum (SR) buffer rather than using the rapid buffering approximation or fixed buffering [27,30]. Experiments suggest the existence of non-junctional DHPR (10–20%), located on the external sarcolemma and not forming the release sites with RyR2 [37,38].

#### 2.1.2. Ryanodine Receptor Type-2 Model

The 2-state ryanodine receptor model incorporates cytosolic calcium-dependent and luminal calcium-dependent gating as described previously with only a small modification to the luminal dependence function to match spark characteristics [27]. The states are with the transition rate $k_{ryr}^{+}$ being increased by subspace Ca^2+^ (${\left[{\mathrm{Ca}}^{2+}\right]}_{ds}^{i}$) and junctional SR Ca^2+^(${\left[{\mathrm{Ca}}^{2+}\right]}_{jsr}^{i}$) and $k_{ryr}^{+}$ being a constant. The RyR2s are arranges in a cluster and display coupled gating as using our previous formulation [27].

(1) $$C\begin{array}{c} \overset{k_{ryr}^{+}}{\to }\\ \underset{k_{ryr}^{-}}{\leftarrow } \end{array}O$$

The opening and closing of individual calcium channels in the CRU are dependent on the local environmental conditions. The RYR2 channels are represented by a 2 state Markov model, where the opening transition rate is dependent on the calcium concentration in both the dyad subspace formed at the interface with the T-tubule and the junctional SR (jSR). The jSR is represented by an additional compartment in the model, which accounts for the limited diffusion of calcium from the non-junctional SR (NSR) found throughout the cell into the substructures at the CRU interface. 

*CaM* has been shown in experiments to associate with RyR2 and modulate its open probability and the IC50 values for *CaM* binding to RyR2 varies with disease causing *CaM* variants [32]. However, the RyR2 open probability has been observed to be higher in CPVT4 causing *CaM* variants but not in LQT14 variants [32]. Furthermore, physiological concentrations of *CaM* lowered RyR2 open probability in failing human ventricular myocytes where the RyR2 was phosphorylated but not healthy human myocytes [39]. This suggests that the interaction of *CaM* and RyR2 is more complicated than simple association or Ca^2+^ binding. For these reasons, in this study of LQT14, the interaction of RyR2 and *CaM* was not included and left for future studies.

#### 2.1.3. L-Type Calcium Channel Gating

**Figure 3:**
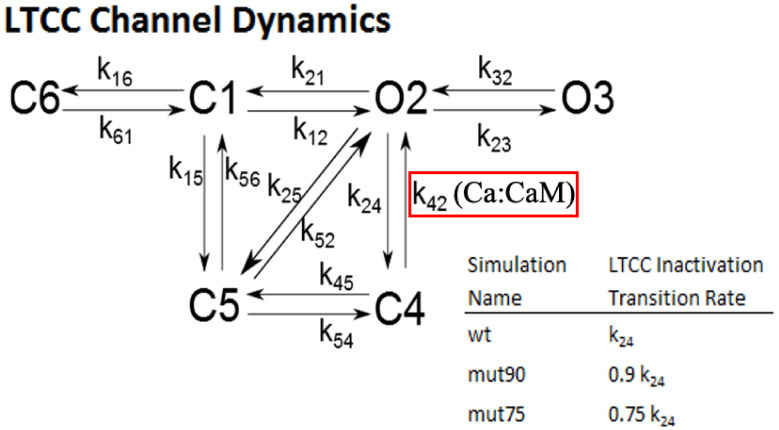
LCC 6 state model. The six states of a single L-type calcium channel (LCC). The transition from the closed state of C1 to the open state O2 is dependent on the transmembrane voltage. The channel inactivation to state C5 is dependent on voltage, but in the CRU, closing is dominated by the *Ca*:*CaM*-dependent transition to C4 (red box). By altering the transition rate to C4, subtle deficiencies in *CaM* inactivation can be modeled. The default value of k_24_ is 8 s^−1^µM^−1^.

The dynamics of the voltage gated LCC are more complex and are represented by the experimentally constrained six state Markov model shown in Figure 3 that was developed by Sun and co-workers and used in our previous work [34,40,41]. The transition rate between most states is dependent on the transmembrane voltage. At resting potential, channels are overwhelmingly likely to be in the closed state C6 and transition to C1 as the membrane potential increases. The transition to O2 occurs when the membrane polarity is reversed, with stronger depolarizations driving the channel into the O3 state. The thermodynamic transition rate from O2->C1 is very small, so LCC inactivation occurs primarily by 2 routes. The voltage-dependent inactivation (VDI) is represented by the transition O2->C5, and the *CaM*-dependent inactivation (CDI) by O2->C4. During CICR, the concentration of calcium in the dyad is 100-fold more than in the myoplasm activating CDI.

**Figure A1:**
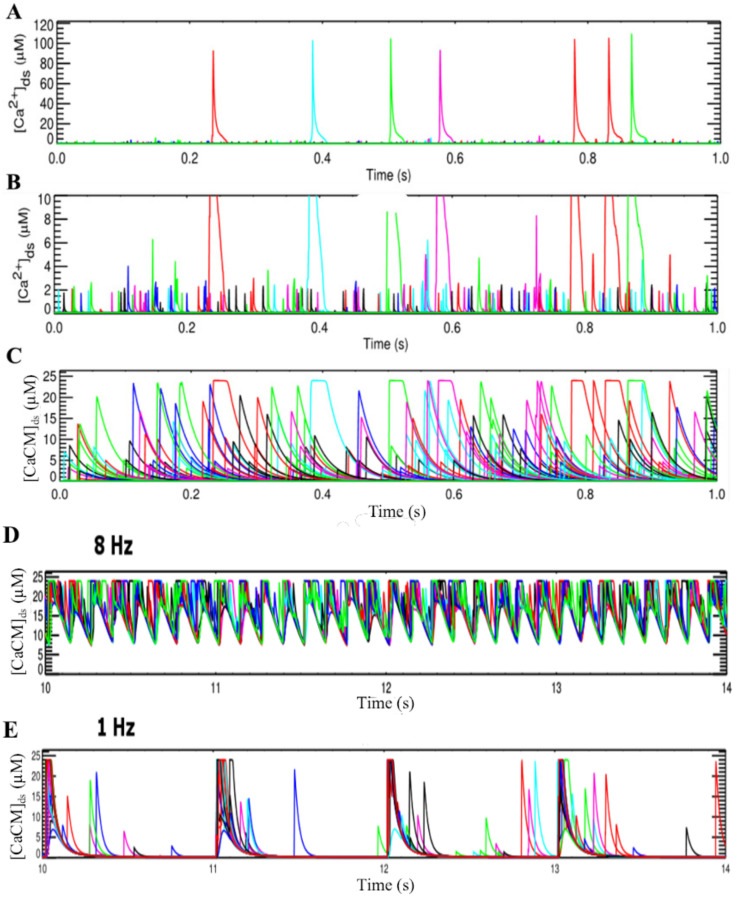
Calcium in the subspaces during (**A**) Ca^2+^ sparks and (**B**) Ca^2+^ quarks, (**C**) The amount of Ca^2+^:*CaM* complex in the subspace during spontaneous Ca^2+^ sparks and quarks at rest. The amount of Ca^2+^:*CaM* complex in the subspace is greater during (**D**) pacing at 8 Hz compared to (**E**) pacing at 1 Hz pacing leading to increased Ca-dependent inactivation of the L-type channel.

The endogenous buffer *CaM* bound to Ca^2+^ (*Ca*:*CaM*) is the effector for Ca^2+^-dependent inactivation (CDI) of L-type Ca^2+^ channel. The calcium-free *CaM* is called apo calmodulin (apo-*CaM*) with two homologous domains, known as lobes [42]. For each apo-*CaM* molecule, there are four different calcium binding sites: two at the E-F hand motifs of the N-terminal (N-lobe) and two at the E-F hand motifs of the C-terminal (C-lobe) of *CaM* (for review see [43]). In L-type Ca^2+^ channels (Cav1.2), CDI is triggered by the binding of Ca^2+^ ions to either the C-lobe or N-lobe of *CaM* with each lobe conferring a fraction of the CDI [44]. The model assumes binding of two Ca^2+^ ions is needed to produce CDI. This differs from the original Sun model, which used the Hill coefficient as 3 based upon earlier data [40]. In addition, the original model did not incorporate the loss of calcium in the subspace due to binding to calmodulin (*CaM*). The bind of Ca^2+^ to *CaM* is modeled using Mass action kinetics as follows:

(2) $$\begin{array}{ll} \frac{d{\left[Ca:CaM\right]}_{dyad}}{dt} & =k_{CaM}^{+}\left({\left[Ca\right]}_{dyad}^{2}\right)\left({\left[CaM\right]}_{T}-{\left[Ca:CaM\right]}_{diad}\right)\\ & -\;k_{CaM}^{-}{\left[Ca:CaM\right]}_{dyad} \end{array}$$

where [*CaM*]*_T_* is the total *CaM* concentration, $k_{CaM}^{+}$ is the binding on rate and $k_{CaM}^{-}$ is the binding off rate with values shown in Table 2. Hence, the contribution of calcium from a small fraction of DHPR I_dhpr,nj_ (15%) was also added. Appendix A Figure A1 shows Ca^2+^ in the subspaces are rest. Ca^2+^ sparks and Ca^2+^ quarks result in increases in the amount of Ca^2+^:*CaM* complex in the dyad. The amount of Ca^2+^:*CaM* complex in the subspace is greater during pacing at 8 Hz compared to pacing at 1 Hz pacing leading to increased Ca-dependent inactivation of the L-type channel. 

The specific mechanisms by which mutations in *CaM* alter the dynamics of LCC inactivation depend on local changes to the way *CaM* interacts with the cytoplasmic domains of the channel. While different mutants can alter the mechanism in unique ways, the net impact on the LCC channel dynamics is to decrease the rate of *CaM*-dependent inactivation. On the scale of the compartmental myocyte model, the LCC interactions with *CaM* are modeled through the transition rate constant k_24_. Lowering the transition rate from O2 to C4 by 10% and 25% simulates mutations in *CaM* which only slightly impact channel function, allowing for the systematic impact on cell function to be studied. The wild type and two mutant CDI models were each simulated for 100 s using a current clamp protocol and determined that a steady train of transients were obtained after 20 s. Simulations were then run 6 times (each with a different seed for the pseudo random number generator) for the wild-type and two mutant CDI models for 30 s, at 1, 2, 4, and 6 Hz stimulation rate. The simulations all converged on a steady train of transients that overlapped. In the figures, data from a single simulation are shown.

To understand how the 10% and 25% reduction in k_24_ might be compared the experimentally observed changes in the Ca^2+^ binding affinity of the *CaM* variants, a steady-state assumption was made about Ca^2+^ binding to *CaM* based on the simulations seen in Appendix A Figure A1. When Equation (2) is solved at steady state, the [*Ca*:*CaM*] is given by

(3) $${\left[Ca:CaM\right]}_{dyad}=\frac{{\left[Ca\right]}_{dyad}^{2}{\left[CaM\right]}_{T}}{{\left[Ca\right]}_{dyad}^{2}+K_{eq}}\;where\;K_{eq}=k_{CaM}^{-}/\;k_{CaM}^{+}.$$

When dyadic Ca^2+^ is assumed to be 10 µM, the ratio of the [*Ca*:*CaM*]*_myo_* of the variant to the wildtype yields the factor modifying k_24_ shown in Table 1. It is important to note that the simulations were not performed to quantitatively predict the consequences of a particular variant.

Living rats have heart rates of 330 to 480 beats per minute which translates to 5.5 to 8 Hz (https://web.jhu.edu/animalcare/procedures/rat.html last accessed 19 December 2022). However, in experimental studies included those that explore CPVT4 and LQTS14 isolated myocytes are stimulated at 1, 2, 3, 4, and other pacing rates. The simulations are performed at the rates used in experiments as well as physiological rates. Interestingly, simulations of wildtype rat ventricular myocytes paced at 8 Hz and observed electrical and Ca^2+^ alternans which are abolished when β-adrenergic stimulation is added [34]. It is possible to simulate human or Guinea pig EC coupling and repeat these simulations. However, these are left for future studies as those models are being developed. However, many of the experimental studies on the effect of *CaM* variants have been performed in rodents, which is the reason this current study was performed in a model of rat ventricular myocyte EC coupling. Simulations at pacing rates 1 Hz and 2 Hz were performed because these stimulation rates are often used in experiments. Simulations at 4 Hz represent the physiological basal stimulation rate in the living rat. The experiments were repeated in a model of beta-adrenergic stimulation at 6 Hz pacing to reflect the functional remodeling which occurs as a result of increased cardiac stress. In this state, the calcium current through the LCC and SERCA channels is increased by a factor of 1.2 and 1.3, respectively. Beta-adrenergic stimulation was also performed at 1, 2, and 4 Hz to show the enhancement in Ca^2+^ loading compared to control simulations that is sometimes studies in experiments.

Several important emergent measures of cellular phenotype are simulated by the model and arise from the independent regulation of calcium channels in CICR. The cyclic increase in calcium concentration in the bulk compartments of the myoplasm is determined by the diffusion of calcium from each of the 20,000 dyad subspace volumes. The subsequent loading of calcium from the bulk cellular volume to the NSR occurs through SERCA channels, and the concentration is also reported by the simulation. Additionally, the flow of potassium, sodium, and calcium ions contribute to the transmembrane voltage and impact the magnitude and duration of the action potential. Additionally, the individual flow through each CRU can be used to determine the independent contributions of the LCC and RYR2 dynamics. When summed, they provide a measure of the contribution to the transmembrane calcium current by the LCC and flux through RYR2 channels. Additional contributions to the calcium current come from the bidirectional Na^+^-Ca^2+^ exchanger (NCX) channels (I_ncx_), which play an important role in restoring the cell to its resting potential. Together, these variables can be used to investigate the underlying mechanism leading to an arrhythmia prone phenotype.

### 2.2. Numerical Methods

The system of ordinary differential equations comprising the model is solved using the explicit Euler method. The simulation uses no-flux boundary conditions based upon the assumption that there was not a significant gradient across cells at the border of the simulation. The adaptive timestep (10–100 nanoseconds) is required for numerical stability and is also necessary to capture the fast and stochastic gating of DHPR (dihydropyridine receptor) and RyR2 channels. Each release site uses a different sequence of pseudo-random numbers to control channel gating. These Monte Carlo simulations are computed on Fermi-GPGPU cards, with pseudo-random numbers derived from the Saru pseudo-random number generator on GPGPU provided by Asfar et al. [45]. When the channel fires, a smaller time-step is selected; first to ensure numerical stability, second to limit maximum 10% of the CRUs having state changes to occur at a specified time [46,47]. Channel gating depends upon the local dyadic subspace (Ca^2+^). The myoplasmic calcium indirectly links all the release sites through modulation of the subspace (Ca^2+^). Thus, the fraction 10% was selected so that the amount of calcium release does not change much in the bulk myoplasm of the system so that these CRUs are assumed to experience the same bulk myoplasmic calcium. Model code is available in the Supplementary Material (File S1).

### 2.3. Statistical Analysis

To ensure that the simulations were consistent each simulation was run 6 times with different starting seed values for the pseudo random number generator. Simulations were compared to make sure that the results were similar across simulations. A representative simulation was chosen to display in the figures. The model generates an intractable amount of Ca^2+^ spark data for the 20,000 Ca^2+^ release units for analysis. To address this, for the Ca^2+^ spark analysis, data from the last second of each simulation were retained for 10% of the 20,000 Ca^2+^ release units (2000 CRUs). The data were processed to calculate spark amplitudes, spark duration, spark frequency, and spark time to peak using a program created in Fortran. For this calculation a spark was considered to start with the dyadic space Ca^2+^ rose above 25 µM. A spark was assumed to terminate when the dyadic space Ca^2+^ subsequently fell below 5 µM. Microsoft Excel was used to calculate the means for these properties and the two-sided student’s *t*-test comparing the means under the assumption of different variances and sample sizes. 

## 3. Results

### 3.1. Reduction of L-Type Calcium Channel CDI Leads to Abnormal EC Coupling

**Figure 4:**
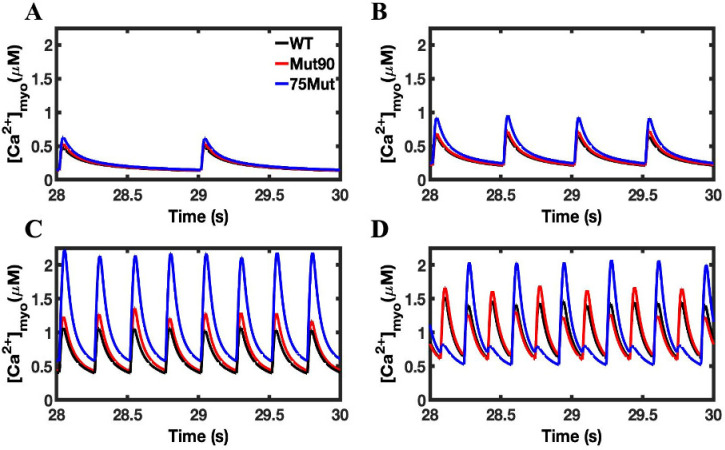
Cellular calcium concentration of cardiac myocytes: (**A**) 1 Hz pacing, (**B**) 2 Hz pacing, (**C**) 4 Hz pacing, (**D**) 6 Hz pacing. The cardiac myocyte simulations of decreased CDI (75% of transition rate—blue and 90% of transition rate—red) show an increase in cellular calcium over the wild-type cells (black). The effect becomes exaggerated with increased pacing resulting in Ca^2+^ alternans. Shown are representative simulations out of six repeated simulations with different random number seed values in each case. The different simulation traces for each condition overlap at steady-state pacing.

**Figure 5:**
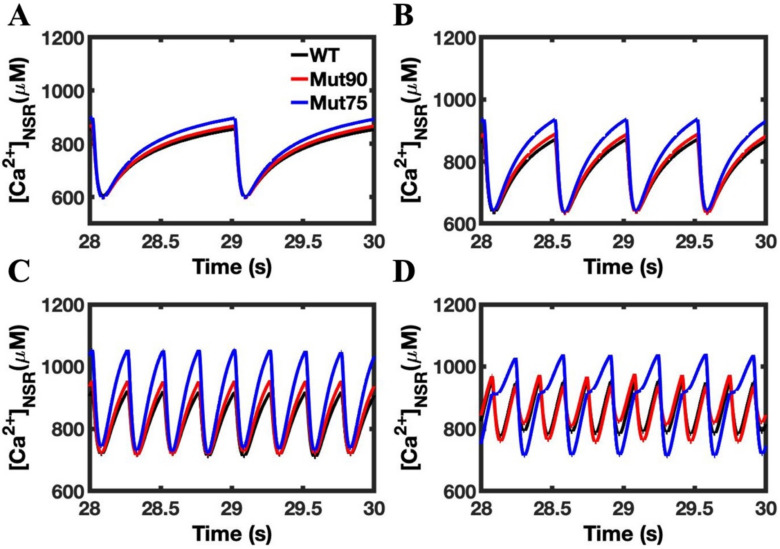
Calcium concentration in the NSR in cardiac myocytes: (**A**) 1 Hz pacing, (**B**) 2 Hz pacing, (**C**) 4 Hz pacing, (**D**) 6 Hz pacing. The mutant simulations CDI (75% of transition rate—blue and 90% of transition rate—red) of the cardiac myocyte show an increase in calcium loading in the SR compared to wild type (black). Shown are representative simulations out of six repeated simulations with different random number seed values in each case. The different simulation traces for each condition overlap at steady-state pacing.

**Figure 6:**
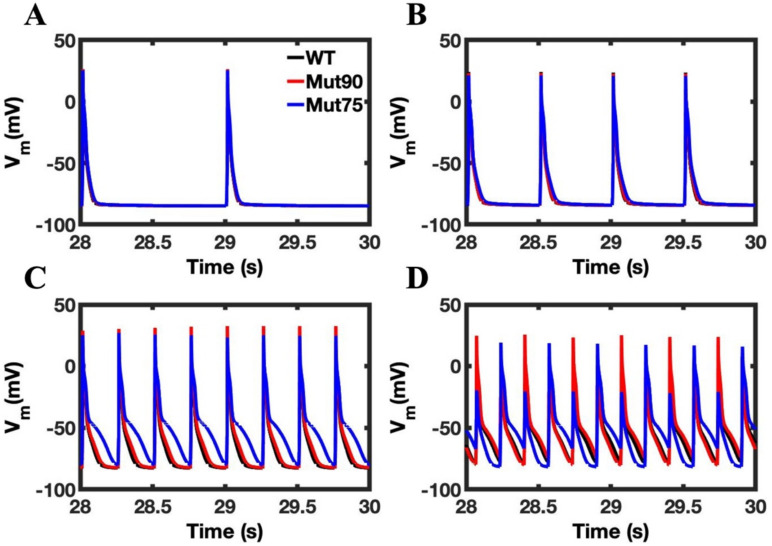
Transmembrane voltage in simulation of cardiac myocytes: (**A**) 1 Hz pacing, (**B**) 2 Hz pacing, (**C**) 4 Hz pacing, (**D**) 6 Hz pacing. Simulations of the wild-type (black) cardiac myocyte and cardiac myocyte with mutant CDI (75% of transition rate—blue and 90% of transition rate—red) show that the transmembrane voltage for the cardiac myocyte displays a slight elongation of the action potential during pacing. APD alternans is seen for the mutant.

The *CaM*-dependent inactivation of LCC plays an important role controlling the amount of Ca^2+^ used to initiate CICR and ensuring the channel is closed prior to the ensuing action potential. The impact of even a slight decrease in the *CaM*-dependent transition probability can be seen in the simulation results in Figure 4, Figure 5 and Figure 6. 

The increase in the peak concentration of cellular Ca^2+^ is seen in both mutants, and the relative increase over the wild-type concentrations is magnified by pacing (Figure 4). An additional impact of pacing is the elevation of the minimum Ca^2+^ concentration level. In the 1 Hz simulation, the mutant cells had time to reach the wild-type diastolic concentrations. As the pacing increased, the minimum cellular Ca^2+^ concentration seen by the cell becomes elevated. For the more severe mutant, the minimum concentration is higher in the 4 Hz pacing (Figure 4C) than the maximum concentration seen for the wild-type cell in the 1 Hz simulation (Figure 4A). Still, the disruption is not severe enough to induce an arrhythmia. The simulations at 6 Hz pacing show a normal train of Ca^2+^ transients for WT (black) but Ca^2+^ alternans developing with the reduction in CDI.

The simulation results for the cardiac myocytes show the mutations impacting the CDI have a similar impact on the NSR calcium dynamics (Figure 5). At the onset of CICR, calcium is rapidly depleted as it diffuses to refill the jSR compartments of each CRU. In the 1 Hz simulations, there is no detectable difference in the rate of depletion, but the mutant simulations show an increased calcium loading during the diastolic state (Figure 5A). Under 2 Hz pacing, the NSR is still fully depleted, and the mutant simulations reaches the same minimum calcium concentration as the wild type (Figure 5B). This is no longer the case when the myocytes are subject to the 4 Hz pacing, where CICR terminates before the NSR can be fully depleted (Figure 5C). In Figure 5D, the refilling of the NSR varies from beat-to-beat with the development of alternans.

**Figure 7:**
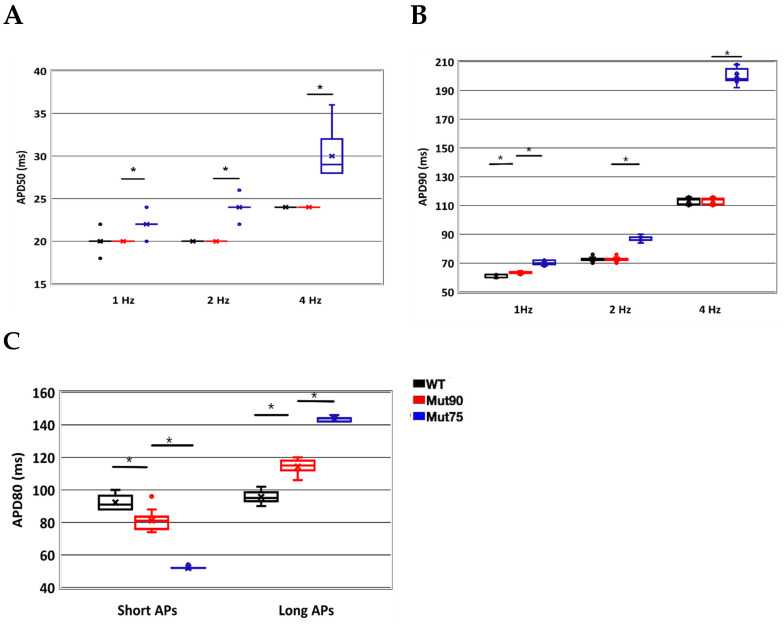
Reduced *CaM* inactivation impact on action potential at different pacing frequencies. (**A**) APD50 for 1, 2, and 4 Hz. (**B**) APD90 for 1, 2, and 4 Hz. (**C**) APD80 for 6 Hz show alternans occurs for the mutant but not wild type. The action potential of cardiac myocytes is elongated in simulations with deficient *CaM* regulated LCC inactivation. (Black—wild type, 75% of CDI transition rate—blue, and 90% of CDI transition rate—red). Shown are representative simulations out of six repeated simulations with different random number seed values in each case. The different simulation traces for each condition overlap at steady-state pacing. The box and whisker chart shows distribution of data into quartiles (boxes indicating middle quartiles separated by a line indicating the mean and lines or whiskers outside the boxes indicating the upper and lower quartiles. The points outside these quartiles are outliers. Statistical significance at the *p* < 0.001 significance level for the student’s *t*-test for equality of means with samples of unknow variance is indicated by the black bars with asterisks.

Despite the changes in calcium loading in both the bulk cell and NSR, the overall impact on the action potential is minimal in the simulations (Figure 6A,B). With 4 Hz pacing, the action potential duration increases a small amount in the 0.90 CDI mutants and a large amount with the 0.85 CDI mutant (Figure 6C). The increase in action potential duration (APD) can be quantified by calculating the average time it takes the transmembrane voltage to return to its diastolic levels over all the depolarizations. In Figure 6D APD alternans is present in the simulated mutants at 6 Hz pacing. The time it takes the cell to rise above 50% and 90% value of the diastolic potential amplitude and return to that point are known as APD50 and APD90, respectively. Figure 7A,B shows that the APD50 and APD90, respectively, for wild type and variant simulated myocytes is significantly increased at 4 Hz and with increased disruption of CDI. Although pacing at 6 Hz yields normal APs for wild type, with the mutants alternans occurs and becomes significantly more severe as the functional defect increases (Figure 7C). APD80 is shown for the long and short action potentials instead of APD90 because the APs at 6Hz in Figure 6D do not always return to 90% repolarization.

**Figure 8:**
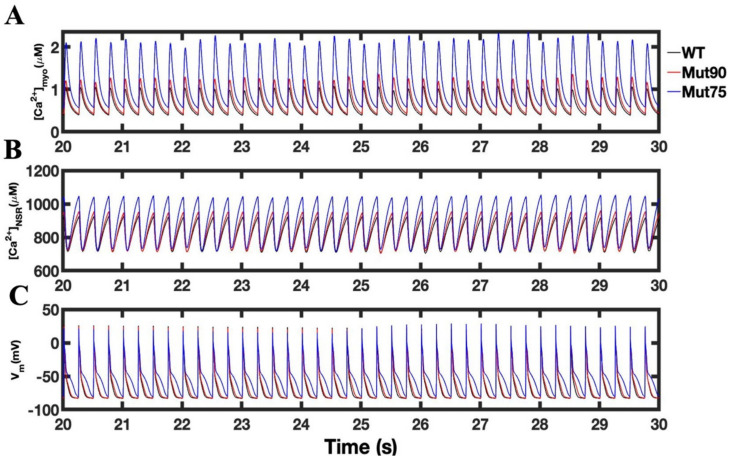
The cardiac myocyte at 4 Hz pacing for 100 s shows that the simulation reaches steady state after 20 s. (**A**) Myoplasmic calcium. Pictured here is the simulation from 20 to 30 s. (**B**) SR calcium. (**C**) Transmembrane voltage. Shown are normal (black) and mutant cells with diminished *CaM*-dependent LCC inactivation. (Red—wild type, 75% of CDI transition rate—blue, and 90% of CDI transition rate—red). Shown are representative simulations out of six repeated simulations with different random number seed values in each case. The different simulation traces for each condition overlap at steady-state pacing.

On a longer timescale, a 30 s simulation at 4 Hz pacing is shown in Figure 8, the magnitude of impact of mutations on the cellular and NSR calcium concentrations is clear (Figure 8A,B). During 4 Hz pacing, all cells respond by with an increased calcium concentrations in the bulk myoplasm and NSR, but the mutants do so with and increased rate and scale (Figure 8C). However, despite these differences in calcium concentration, the impact of the *CaM* mutations on the action potential is less pronounced. In the simulation for a given cell type (wild type and mutant), the difference in action potential from beat to beta is negligible after 20 s because all simulations are able to achieve a new homeostasis. This is further demonstrated by the uniformity of the cyclic peaks in the final second of simulations. Still, the simulated mutations do push the simulations toward instability, as oscillations in the peak cellular calcium begin to occur for the more severe mutant under 4 Hz pacing. In this condition, the cell has assumed a state on the verge of arrhythmia, indicating a possible threshold of diminished CDI function that is tolerated by the cardiac myocyte. 

### 3.2. Changes in Ca^2+^ Spark Dynamics Underlie Arrhythmogenesis

**Figure 9:**
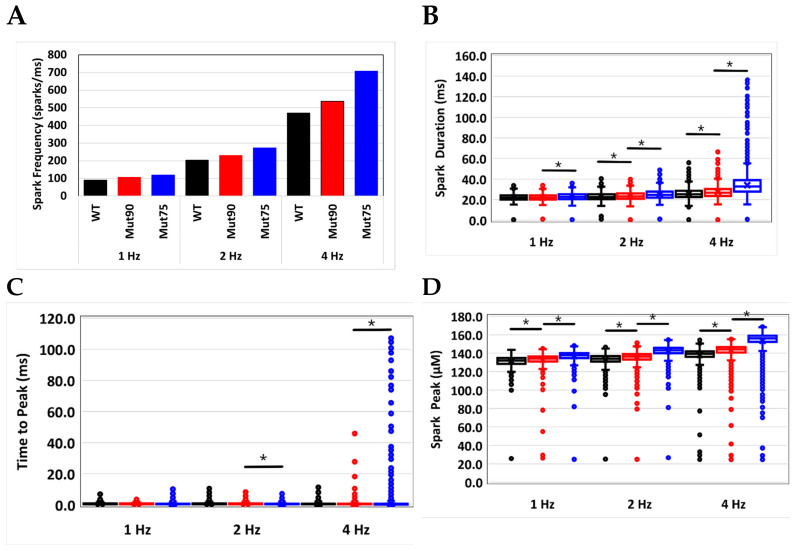
Calcium sparks in the cardiac myocyte at different pacing frequencies recorded for 1 s at steady-state pacing. (**A**) Spark frequency, (**B**) spark duration, (**C**) time to Ca^2+^ spark peak, and (**D**) Ca^2+^ spark peak amplitude. (Black—wild type, 75% of CDI transition rate—blue, and 90% of CDI transition rate—red). The box and whiskers plots show the middle two quartiles with boxes, the upper and lower quartiles with lines, the mean with an ×, and outliers with points. Statistical significance at the *p* < 0.001 significance level for the student’s *t*-test for equality of means with samples of unknow variance is indicated by the black bars with asterisks. Shown are representative simulations out of six repeated simulations with different random number seed values in each case. The different simulation traces for each condition overlap at steady-state pacing. The number of data points for each simulation is equivalent to number of sparks is 1000 × the values shown in (**A**). Only the data for 10% of the 20,000 Ca^2+^ release sites are not shown because it is impractical to store the large amount of data.

Occasionally, the RYR2 channel will spontaneously transition to the open state to produce a non-coordinated release of Ca^2+^ from the CRU. Known as a Ca^2+^ spark, the Ca^2+^ release can trigger larger changes in the ionic homeostasis and promotes arrhythmia when occurring spontaneously at the wrong time, and the impact of the small Ca^2+^ release is compounded when sparks increase in frequency, duration, and rate. As seen in Figure 9A, diminished *CaM* inactivation leads to an increase in spark frequency over the wild-type model. Table 3 shows the mean values for the Ca^2+^ spark properties. The spark duration during pacing ranges between 89.6–707.9 sparks per ms which translates to up 100,000’s of Ca^2+^ sparks per second. With such a large sample size the standard deviation will be small and the difference of the means will show statistical significance [48]. For example, with even a 1% difference in means (with mean = 200) and the standard deviation equal to the mean, the sample size would simply need to be ~160,000 to achieve statistical significance at the *p* < 0.001 level. Hence, statistical significance is not shown. At 4 Hz the average Ca^2+^ spark duration is increased significantly with the mutants and increases pacing frequency (Figure 9B). The spread of the spark durations increases at 4 Hz when arrhythmia occurs consistent with experiments and other simulation studies. The simulations also predict a delayed average time to peak with increasing frequency and severity of mutation (Figure 9C). Similar to spark duration the dispersion of the time to peak increases under arrhythmic conditions. The average peak spark magnitude increases with the severity of CDI disruption for each pacing frequency (Figure 9D) and more outliers are observed under arrhythmic conditions. Pacing at 1 Hz produces a greater average spark duration for the wild-type myocyte over the mutant models. As the rate of pacing increases, the severity of the mutations causes increase dispersion of Ca2^+^ spark properties. When coupled with the increased spark frequency of the mutant myocytes, the total spontaneous Ca2^+^ release could make significant contributions to developing an arrhythmia. Rat ventricular myocytes harboring *CaM* mutants showed a decreased loading of the SR produced by an increased spark rate [32]. Since the cells were not undergoing pacing CICR, the increased spark rate is attributed to altered *CaM* binding to the RYR2 channels. Since *CaM* interactions with RYR2 was not modeled in the current simulations, the increase in calcium sparks caused by diminished CDI further increases the potential for EC coupling disruption.

### 3.3. Beta-Adrenergic Stimulation Potentiates Ca^2+^ Overload in Myocytes with Reduced CDI

**Figure 10:**
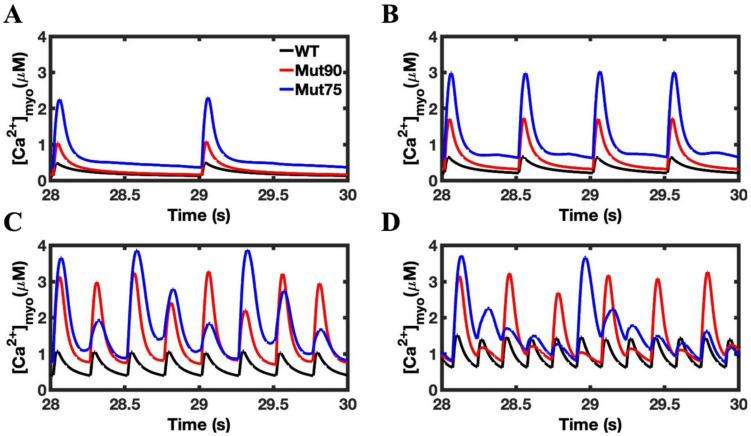
Cellular calcium for beta-adrenergic stimulated cardiac myocytes: (**A**) 1 Hz pacing, (**B**) 2 Hz pacing, (**C**) 4 Hz pacing, (**D**) 6 Hz pacing. The mutant simulations CDI (75% of transition rate—blue and 90% of transition rate—red) of the cardiac myocyte show that the impacts on cellular calcium concentration of mutant cardiac myocytes is exaggerated under beta-adrenergic stimulation compared to wild type (black). Shown are representative simulations out of six repeated simulations with different random number seed values in each case. The different simulation traces for each condition overlap at steady-state pacing.

**Figure 11:**
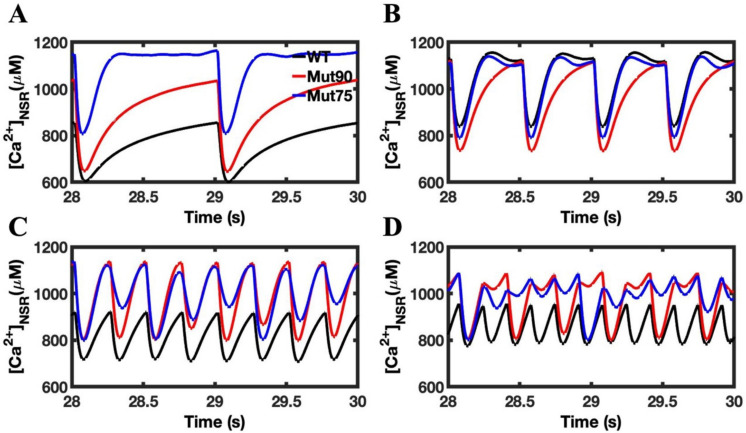
NSR calcium in beta-adrenergic stimulated cardiac myocytes: (**A**) 1 Hz pacing, (**B**) 2 Hz pacing, (**C**) 4 Hz pacing, (**D**) 6 Hz pacing. The wild-type cardiac myocytes (black) show consistent morphology with increased pacing. The mutant simulations CDI (75% of transition rate—blue and 90% of transition rate—red) of the cardiac myocyte show that in beta-adrenergic cardiac myocytes, the NSR calcium dynamics of CDI mutants become severely disrupted in response to pacing. Shown are representative simulations out of six repeated simulations with different random number seed values in each case. The different simulation traces for each condition overlap at steady-state pacing.

**Figure 12:**
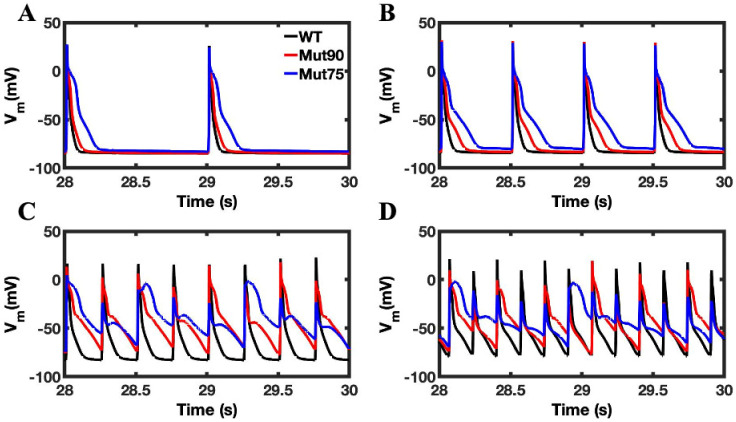
Transmembrane voltage in beta-adrenergic stimulated cardiac myocytes: (**A**) 1 Hz pacing, (**B**) 2 Hz pacing, (**C**) 4 Hz pacing, (**D**) 6 Hz pacing. The wild-type cardiac myocytes (black) show consistent morphology with increased pacing. The mutant simulations CDI (75% of transition rate—blue and 90% of transition rate—red) of the cardiac myocyte show that during pacing, the ability of mutant beta-adrenergic cardiac myocytes to repolarize is diminished and leads to arrhythmia. Shown are representative simulations out of six repeated simulations with different random number seed values in each case. The different simulation traces for each condition overlap at steady-state pacing.

Beta-adrenergic stimulation induces functional changes in the cardiac myocyte in response to stress or exercise. Under normal conditions, the increase in calcium flux through the LCC and SERCA allow the cardiac myocyte to cope with the increased demands of higher frequency pacing. The results for the beta-adrenergic simulations are shown in Figure 9, Figure 10 and Figure 11, where the deleterious impact of diminished CDI is clearly shown by the disruption to both calcium dynamics in the myoplasm (Figure 10) and in the NSR (Figure 11) and the action potential (Figure 12) when pacing is applied. The increased flow of calcium through the LCC in response to beta-adrenergic stimulation is enhanced by the impact of the diminished CDI. In response to pacing, the exaggeration leads to the development of an arrhythmia, where the regular cyclic peak in cellular calcium concentration is lost.

The other impact of beta-adrenergic stimulation is to increase the rate calcium is loaded into the SR through the SERCA pumps. In the more severe of the CDI mutants, the increased loading in the 2 Hz pacing results in the NSR never depleting during CICR. The increased calcium concertation increases the open probability of the RYR2 channels and underlies the increase in APD seen in Figure 11. Even in the less severe mutant, 4 and 6 Hz pacing induces arrhythmia and causes instability in the NSR calcium dynamics.

In the wild-type simulations of beta-adrenergic stimulated cardiac myocytes, the calcium concentrations in the myoplasm (Figure 10D) and the NSR (Figure 11D) reach similar levels as seen in the mutant simulations. However, instead of devolving into arrhythmia, the wild-type simulations are able to balance the increased calcium load to maintain depolarization during the action potential (Figure 12D). 

While the magnitude of the action potential is diminished in the 6 Hz wild-type simulation, the membrane still depolarizes. Conversely, both mutant simulations produce arrhythmic behavior in the 6 Hz pacing of beta-adrenergic cardiac myocytes, developing into a phenotypic state of period-doubling (alternans) characteristic of Ca^2+^ overload in CPVT4. As seen in Figure 10D, Figure 11D and Figure 12D, EC coupling also breaks down during 2 Hz pacing due to the increased NSR calcium loading in the more severe mutant. The increased calcium concentration increases the refilling of the jSR and increases the open probability of the RYR2 channels. The mut75 action potential is elongated to produce a LQT14 phenotype while the mut90 action potential only deviates slightly from the wild type. The model demonstrates how different emergent phenotypes can arise out from a single underlying deficiency in molecular function. 

**Figure 13:**
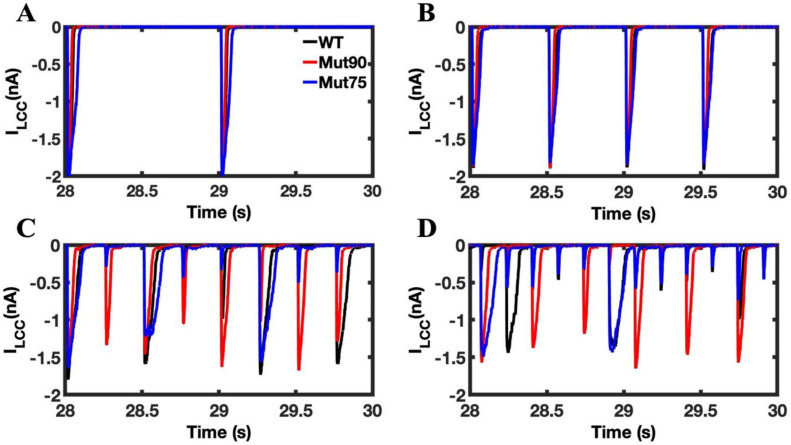
LCC calcium current in cardiac myocyte simulations under beta-adrenergic stimulation: (**A**) 1 Hz pacing, (**B**) 2 Hz pacing, (**C**) 4 Hz pacing, and (**D**) 6 Hz pacing. The wild-type cardiac myocytes (black) show consistent morphology with increased pacing. The mutant simulations CDI (75% of transition rate—blue and 90% of transition rate—red) of the cardiac myocyte show that the differences in the Ca^2+^ current through the LCC are undetectable in the cardiac myocyte simulations, but the impact of reduced CDI increased the current during pacing in beta-adrenergic stimulation. Shown are representative simulations out of six repeated simulations with different random number seed values in each case. The different simulation traces for each condition overlap at steady-state pacing.

An advantage of the computational model is that it allows the underlying mechanism of the arrhythmia to be probed in detail. The calcium current through the LCC was totaled across all 20,000 CRUs to produce the plots in Figure 13. The importance of CDI on controlling the initial flow of calcium during in seen in the failure of mutant simulations to maintain a tight window of current flow into the cell. In the simulations without beta adrenergic stimulation, there is virtually no difference in the current through the LCC. However, during beta-adrenergic stimulation, the diminished inactivation increases the length of time calcium enters the cell. This effect is enhanced by pacing, but in all simulations LCC inactivation is relatively rapid occurs long before the onset of the following action potential. Still, the small increase in the influx alters the dynamics of CICR by increasing the open probability of RYR2 channels on the SR membrane. 

**Figure 14:**
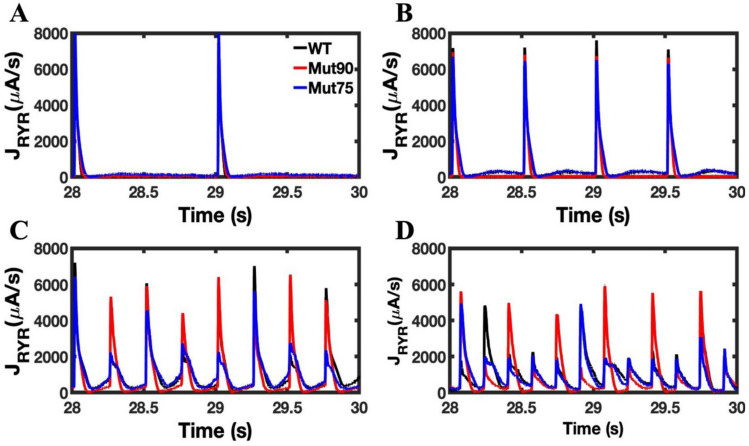
Flux through RYR2 channels in cardiac myocyte simulations under beta-adrenergic stimulation: (**A**) 1 Hz pacing, (**B**) 2 Hz pacing, (**C**) 4 Hz pacing, (**D**) 6 Hz pacing. The wild-type cardiac myocytes (black) show consistent morphology with increased pacing. The mutant simulations CDI (75% of transition rate—blue and 90% of transition rate—red) of the cardiac myocyte show that the total calcium flux released through the RYR2 channels of 20,000 CRUs displays an increase in calcium sparks during beta-adrenergic stimulation. An additional increase in the open probability of RYRs comes from the higher calcium concentration in the dyadic subspace from their prolonged APD. Shown are representative simulations out of six repeated simulations with different random number seed values in each case. The different simulation traces for each condition overlap at steady-state pacing.

The increase in open probability of the RYR2 channels causes a small increase in the Ca^2+^ flux from the SR. The total calcium flux through all the RYR2 channels in the 20,000 independent CRUs is provided in Figure 14. In the cardiac myocyte simulations without beta adrenergic stimulation, the magnitude of the Ca^2+^ release is increased in the CDI mutants while the duration of release remains the same. The increased window of calcium current through the LCC initiates CICR in an increased number of RYR2 channels but does not impact the transition to a closed state. However, when beta-adrenergic stimulation is applied the increased loading in the SR elongates the Ca^2+^ release into the dyad subspace. During pacing, these small changes accumulate and ultimately interrupt the myocyte’s ability to regulate the transmembrane voltage through the Na^+^-Ca^2+^ exchanger.

**Figure 15:**
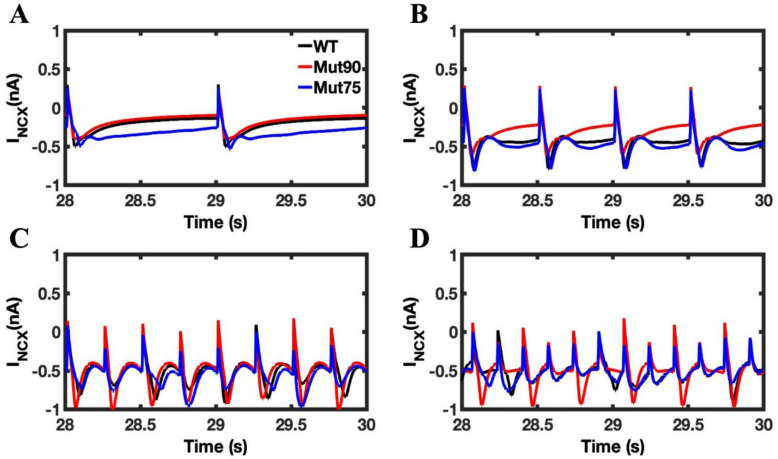
Na^+^-Ca^2+^ exchanger calcium current in myocyte simulations under beta-adrenergic stimulation. (**A**) Na^+^-Ca^2+^ exchanger current at 1 Hz pacing. (**B**) Na^+^-Ca^2+^ exchanger current at 2 Hz pacing. (**C**) Na^+^-Ca^2+^ exchanger current at 4 Hz pacing. (**D**) Na^+^-Ca^2+^ exchanger current at 6 Hz pacing. The wild-type cardiac myocytes (black) show consistent morphology with increased pacing. The mutant simulations CDI (75% of transition rate—blue and 90% of transition rate—red) of the cardiac myocyte show that the currents through the Na^+^-Ca^2+^ exchanger in the cell membrane becomes destabilized in mutant beta-adrenergic stimulation, as the increased calcium prevents the current to reach the initial diastolic levels.

The current through the Na^+^-Ca^2+^ exchanger in the cell membrane is shown in Figure 15. During the action potential, the normal flow of calcium out of the cell in reversed to export the large influx of sodium and is shown by positive peak in Na^+^-Ca^2+^ exchanger current. This reversal in flow is necessary to restore the action potential in the cell membrane. However, the accumulated impact of the increased CICR drives up the calcium concentration in the myoplasm and reduces gradient responsible for driving the ion the exchange. As seen in the 1 Hz simulations, the impact of the CDI mutation on is to prolong the time restore the Na^+^-Ca^2+^ exchanger current to the diastolic level. In the beta-adrenergic stimulated myocytes, the increase is magnified, and the effect accumulates in the more severe mutant and is accelerated by pacing. Once the cumulative impact reaches a threshold, the cell is no longer able to reverse current in response to the action potential. The ability to maintain the transmembrane potential is lost and the rhythmic pacing of the cell deteriorates into arrhythmia. 

The overall result of a small change to the rate of CDI leads to the rapid formation of arrhythmia, as the high cellular concentration of calcium interrupts the ability to regulate the transmembrane voltage through the Na^+^-Ca^2+^ exchanger. Once the calcium concentration becomes too large, the channels are not able to efficiently reverse direction to export sodium. The mechanism feeds into itself, as the increased flow through an individual CRU promotes larger releases through loading of calcium into the NSR, until the cell reaches a state where the regulatory mechanism is overcome. Arrhythmias associated with CPVT4 typically have altered RYR2 function but can also occur by diminished function of other channels which influence the concentration of ions in the cardiac myocyte. The movement of calcium through the cellular compartments creates an interconnected relationship between channel dynamics and underscored the difficulty in identifying the mechanism associated with a disease phenotype only detectable at the tissue level.

## 4. Discussion

The simulation study presented here explored how mutations to N98S and D96V that result in reduced CDI corresponding can lead to abnormal EC coupling and therefore arrhythmia. As the CDI was reduced by a factor of 0.9 (N98S) and 0.75 (D96V) in the variants the action potential duration increased a small amount. This is consistent with clinical findings that the QT prolongation in patients with the N98S variant is small [4]. and Ca^2+^ dynamics result in a normal wave train during 1 Hz, 2 Hz, and 4 Hz pacing rates. A pacing rate of 1 Hz and 2 Hz are often used in experimental studies and a 4 Hz pacing rate is the physiological heart rate of rat. Rapid pacing at 6 Hz resulted in abnormal Ca^2+^ dynamics and action potential dynamics in the form of alternans which is precipitated by high level of Ca^2+^ in the ventricular myocyte.

Under beta adrenergic stimulation, pacing at 1 Hz and 2 Hz showed a steady train of Ca^2+^ transients and action potentials with prolonged duration in the mutant simulations. Pacing at 4 Hz under beta adrenergic stimulation produced alternans in both the Ca^2+^ transient and action potentials. Pacing at 6 Hz, resulted in a very irregular train of action potentials and the underlying Ca^2+^ dynamics. Under the rapid pacing simulations Ca^2+^ levels were significantly elevated in the SR and myoplasm consistent with Ca^2+^ overload. For comparison, in a mouse knock-in model the N98S mutant ventricular myocytes showed that beta adrenergic stimulation induced a delay in repolarization and increase L-type Ca^2+^ current in optical and electrical measurement of membrane potential in isolated hearts compared to control [5].

Underlying the arrhythmic behavior was an increase in Ca^2+^ spark frequency, amplitude, and duration. These findings are supported by other studies. Wescott and co-workers also observed in increase in Ca^2+^ spark duration in their simulations of CPVT when arrhythmia was observed [49]. In experiments flecainide reduced spark amplitude, decreased Ca^2+^ wave incidence, and suppressed CPVT symptoms in mice and humans [50,51]. Macquaide and co-workers observed in a joint experimental and modeling study that in sheep cardiac myocytes experiencing atrial fibrillation, myocytes had >50% higher spark frequency with increased spark time to peak and duration [52]. This increase in Ca^2^ spark frequency has also been observed in atrial fibrillation in human atrial myocytes by Hove-Madsen and co-workers [53].

Calmodulin variants have been associated with long QT syndrome 14 (LQT14), catecholaminergic polymorphic ventricular tachycardia type 4 (CPVT4), and idiopathic ventricular fibrillation (IVF), however, arrhythmogenesis has been attributed mainly to either prolonged repolarization (LQT14 phenotype) or increase calcium release from the SR (CPVT phenotype) [2,54]. The recent association of mutations in *CaM* with these two arrhythmic cardiac diseases raises questions about how complex, emergent disease states are classified [1]. The mutations that are strongly associated with LQT14 strongly disrupt the *CaM*-dependent inactivation of LCC and are easy to diagnose using an ECG. However, other mutations in structurally similar locations in *CaM* manifest as the harder to detect CPVT4. Since *CaM* interacts with a range of other proteins in the cell such as the Na^+^ channel and SK channel, even those involved in regulating EC coupling and CICR, it’s possible that some of the *CaM* mutants’ function normally in LCC inactivation and the CPVT4 state arises through other interactions [55,56,57]. Experimental studies have shown that the LQT14 phenotype does not depend on *CaM* interactions with the Na^+^ channel [8].

The prolonged action potential of guinea-pig ventricular myocytes expressing mutant *CaM* associated with LQT14 shown in Figure 1 (in Reference [31]) displayed a much more severe impact on the action potential and cellular calcium. The impacts of those mutations on the *CaM* inactivation of LCC would clearly alter the electrical signature on the tissue scale and make the increased chances for arrhythmia easily identifiable. In fact, and experimental optical mapping study in heart by Němec and co-workers suggested that the abnormal Ca^2+^ dynamics associated with CPVT, can also be associated with the T-wave lability found in LQT [58]. The underly defect in the Ca^2+^ dynamics is the Ca^2+^ overload in the myocyte for both LQT and CPVT4 [59,60]. For less severe *CaM* mutations, the deviation from the wild-type action potential would be difficult to detect using available diagnostic methods, and a mutation which only fractionally decreases the *CaM*-dependent LCC transition to a closed state would avoid detection in the unstimulated heart. While the phenotypic impact of less severe *CaM* mutations is not as easily detectable, the altered dynamics of the cardiac myocyte increase the susceptibility to arrhythmia.

**Figure 16:**
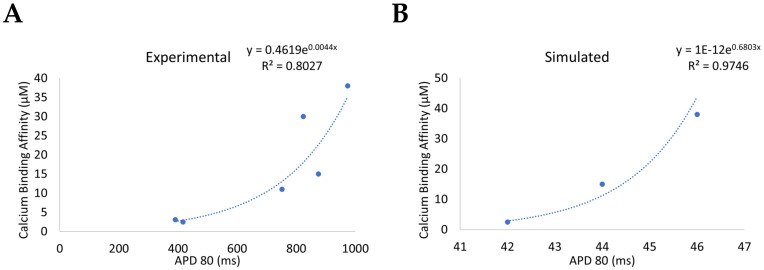
APD80 and calcium binding affinity show a monotonic non-linear relation correlation coefficient. (**A**) Experimental data of APD80 at 0.5 Hz pacing in Guinea pig ventricular myocytes from [1,31,61]. (**B**) Simulated rat ventricular myocyte data of APD80 at 1 Hz pacing from this study.

The action potential duration (APD80) and the Ca^2+^ binding affinity changes for the different *CaM* variants has been measured experimentally by others (see [1,31,61]). Figure 16A shows the relationship between APD80 (in experiments on Guinea pig ventricular myocytes paced at 0.5 Hz) and K_D_ is a monotonically increasing non-linear function with a high correlation coefficient. The simulations in Figure 16B using a rat ventricular myocyte model from this current study paced at 1 Hz shows a similar monotonically increasing trend where calcium binding affinity is approximated by the inverse fractional change. The binding affinities were estimated using the assumption of rapid equilibrium and that subspace calcium remains elevated at 10 µM. In this work, changes to the rate constant were implemented and specific variants were bit simulated.

The simulations clarify that Ca^2+^ overload is the mechanism behind the overlapping phenotypes of the arrhythmia and provide a way of differentiating how they arise. The simulations show that even a small change to the rate of *CaM* regulated inactivation of the LCC promotes an arrhythmia prone state. Increased calcium loading in the bulk myoplasm, and SR is exaggerated through pacing, as is the spark rate and intensity. The altered ionic homeostasis increases the likelihood of disrupting the EC coupling cycle by a mechanism similar to CPVT4, despite the originating from disrupted function associated with LQT14. A greater understanding of conditions which give rise to complex genetic diseases such as these in enhanced through modeling the emergent impact of altering individual functional interactions. 

In the cardiac ventricular myocyte CDI is a complex phenomenon conferred by the interactions of *CaM* with Ca^2+^ and the L-type Ca^2+^ channel. The two lobes of *CaM* each have two Ca^2+^ binding sites and both of the are involved in CDI [44]. *CaM* variants associated with LQT14 affect Ca^2+^ binding sites in both lobes. The current model does not differentiate between the contributions of the two lobes and their combined contributions to CDI. This has been left for future studies. The experimental literature gives a wide range of values of the Ca^2+^ binding affinity of *CaM* ranging from 2.5 to 150 µM when interacting with the L-type Ca^2+^ channel and from 0.03 to 4.0 µM when interacting with the RyR2 [6,8,26,27,28,29,30,31,32,33]. *CaM* seems to respond to the high Ca^2+^ inside the CRU during a Ca^2+^ spark (>100 µM) as well as the bulk myoplasmic Ca^2+^ levels seen in the myoplasm (1 µM). The varied values observed experimentally might all be valid with the value depending on the experimental (or cellular) environment of *CaM*. Understanding these complex issues will require integration of new experimental and modeling studies.

Previous computational modeling studies have studied the mechanisms LQT14. Tadross et al. developed a model of Ca^2+^-dependent inactivation by calmodulin that demonstrated the binding and unbinding of calmodulin from the L-type Ca^2+^ channel as a mechanism of complex decoding of the Ca^2+^ signal [62]. Lai and co-workers created at two-state allosteric model of calmodulin and it’s binding to different molecular targets [63]. The present study did not go into such detail and instead considered the consequences of reduced Ca^2+^-dependent activation on excitation–contraction coupling, Limpitikul et al. developed a bi-lobal model of Ca^2+^-dependent inactivation in the cardiac ventricular myocyte of the L-type channel based on experimental data to characterize the effects of the D130G variant [64]. They showed the resulting action potential prolongation resulting from this variant. While their methodology provides a way to simulate other variants, it does not explain the mixed phenotype observed from certain variants as explored by this modeling study. Numerous other computational studies have studied LQT resulting from variants in other ion channels with mechanism of the action prolongation differs from this study [65,66,67,68]. For example, Kernik and co-workers studied genetic variants of KCNQ1, Bai and co-workers studied the CACNA1C R858H variant (LQT8), and Sadrieh and co-workers studied variants in I_Kr_ (LQT2). Grandi and co-workers studied the compound mutations involving the slowly and rapidly activating delayed-rectifier potassium currents (I_Ks_ and I_Kr_) and the fast sodium current (I_Na_). 

## 5. Conclusions

Our computational model of the rat ventricular myocyte demonstrates the changes in the features of excitation–contraction coupling in myocytes with wild-type and variant calmodulin. With variants that are attributed to causing LQT14, the myocytes have increase action potential duration and large Ca^2+^ transients due to loss of Ca^2+^-dependent inactivation. These changes are potentiated with beta adrenergic stimulation and are accompanied by the development of Ca^2+^ overload and alternans which is seen during CPVT4. The increased RyR opening due to the increased myocyte Ca^2+^ contributes to this phenotype. Taken together, this confluence of phenotypic features can cause the LQT14 variants causing a change in Ca^2+^-dependent inactivation to appear as if they are causing a CPVT4 phenotype. 

## Figures and Tables

**Table 1:** Disease-associated mutations in *CaM*.

Mutation *	Disease Association	Structural Domain	Activity Change	Functional Change	Ca^2+^ Binding Affinity	Ca^2+^-Dependent Inactivation **	*CaM*1	*CaM*2	*CaM*3
WT	none		none	none	2.5 µM	k_24_			
N54I	CPVT4	EF Hand II adjacent	Small increase in Calcium binding in C-lobe	Significantly increased calcium waves	3.1 µM	0.99 × k_24_	x		
F90L	IVF	EF Hand III adjacent		Mediates interaction with target peptides			x		
D96V	LQT14	EF Hand III	Moderate/High disruption of Calcium binding	Increased AP; depress LTCC inactivation	38.0 µM	0.74 × k_24_		x	
N98S	CPVT4; LQT14	EF Hand III	Significant reduction in calcium binding	RYR2 binding disrupted at low Ca^2+^	11.0 µM	0.92 × k_24_	x	x	
N98I	LQT14	EF Hand III	Reduced calcium binding	Increased RYR open probability				x	
A103V	CPVT4	EF Hand III							x
D130G	LQT14	EF Hand IV	Reduced calcium binding	Reduced RYR2 binding affinity; increased AP; impaired LTCC inactivation	150.0 µM	0.41 × k_24_	x		x
D132E	CPVT4; LQT14	EF Hand IV	Reduced calcium binding					x	
D134H	LQT14	EF Hand IV	Reduced calcium binding					x	
Q136P	CPVT4; LQT14	EF Hand IV	Reduced calcium binding					x	
E141G	LQT14	EF Hand IV adjacent	Reduced calcium binding	Impaired LTCC inactivation			x		
F142L	LQT14	EF Hand IV adjacent	Reduced calcium binding	Reduced RYR2 binding affinity; increased AP; depress LTCC inactivation; Alter energetic coupling in C-lobe conformational dynamics	15.0 µM	0.89 × k_24_	x	x	

* Mutations in calmodulin (*CaM*) have been associated with Long QT Syndrome Type 14 (LQT14), idiopathic ventricular fibrillation (IVF) and catecholaminergic polymorphic ventricular tachycardia type 4 (CPVT4). ** The fraction change to CDI is described by a reduction in the *CaM* bound by Ca^2+^ assuming rapid equilibrium of Ca^2+^ binding to calmodulin. The default value of k_24_ is 8 s^−1^ µM^−1^.

**Table 2:** Calcium buffers.

Variables	Value	Unit
Calmodulin	2.4d1	µM
$k_{CaM}^{+}$	3.0d1	1/(µM.s)
$k_{CaM}^{-}$	7.14d1	1/s

**Table 3:** Ca^2+^ spark properties for mutations in *CaM* at different pacing frequencies.

Frequency	1 Hz			2 Hz			4 Hz		
Variant	WT	Mut90	Mut75	WT	Mut90	Mut75	WT	Mut90	Mut75
Spark Frequency (sparks/ms)	89.6	105.9	119.5	204	229	272.9	470.7	536.5	707.9
Spark Duration (ms)	22.2	22.1	23.1	23.0	23.6	25.0	25.7	27.3	34.1
Time to Peak (ms)	0.78	0.74	0.73	0.75	0.75	0.68	0.71	0.70	1.02
Peak Amplitude (µM)	131.4	133.1	137.0	133.2	135.5	142.3	138.2	143.0	153.5

## Data Availability

The rat ventricular myocyte model was recently published, and the parameters described below are for the normal and HF setting. Please check [34,69] for the detail and description of the parameters of the model.

## Supplementary Materials

The following supporting information can be downloaded at https://www.mdpi.com/article/10.3390/biom13010072/s1, File S1: Model code.
